# What is ‘spiritual health’- a survey of UK Social Prescribers

**DOI:** 10.1093/geroni/igaf122.3134

**Published:** 2025-12-31

**Authors:** Ishbel Orla Whitehead, Amy O’Donnell, Barbara Hanratty

**Affiliations:** Newcastle University, Newcastle upon Tyne, England, United Kingdom; Newcastle University, Newcastle upon Tyne, England, United Kingdom; Newcastle University, Newcastle upon Tyne, England, United Kingdom

## Abstract

Background: Holistic (whole person) health includes spiritual health. Social prescribing is a UK initiative that aims to provide holistic, or salutogenic (health creating) care to patients, utilising community links, beyond that offered in consultations with health professionals. Support may be directed at many aspects of health and wellbeing, including spiritual health. Spiritual health is associated with many other physical, mental and rehabilitation (restorative care) outcomes. However, how social prescribers define ‘spiritual health’ is unknown. To explore what social prescribers understand by the term “spiritual health.” We designed a custom online survey of social prescribers in the UK and asked ‘what does spiritual health mean to you?’ Free text data were subject to thematic analysis using a priori themes from the literature on definitions of spiritual health. One hundred and seventy one social prescribing link workers responded; 153 gave a definition of spiritual health. Twice as many participants described themselves as spiritual than described themselves as religious. A very small minority found the term ‘spiritual health’ to be meaningless. Definitions of spiritual health were categorised into themes of sense of self, peace, meaning and purpose; Connections to others, the world, or a deity; Spiritual or religious practice. Spiritual health appears to be a term with meaning to UK social prescribers, distinct from religiosity. This ‘working definition’ of spiritual health will allow further research into how spiritual health fits within social prescribing in the UK.

